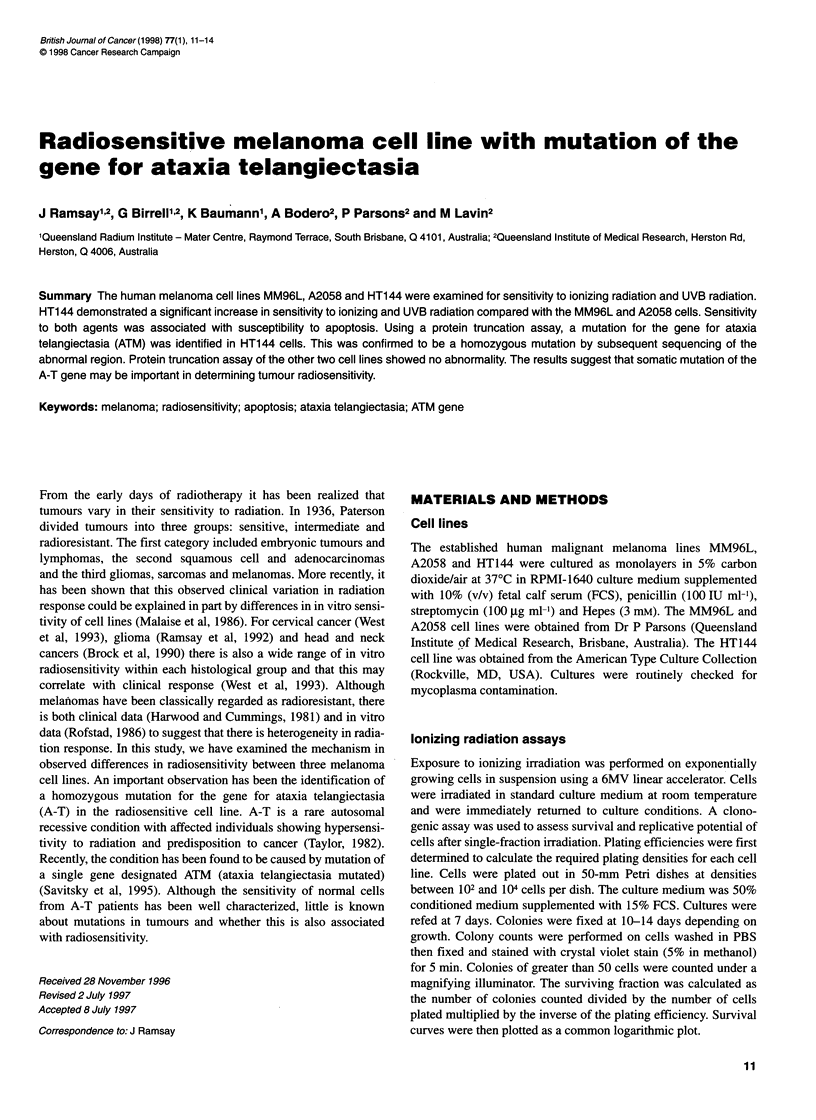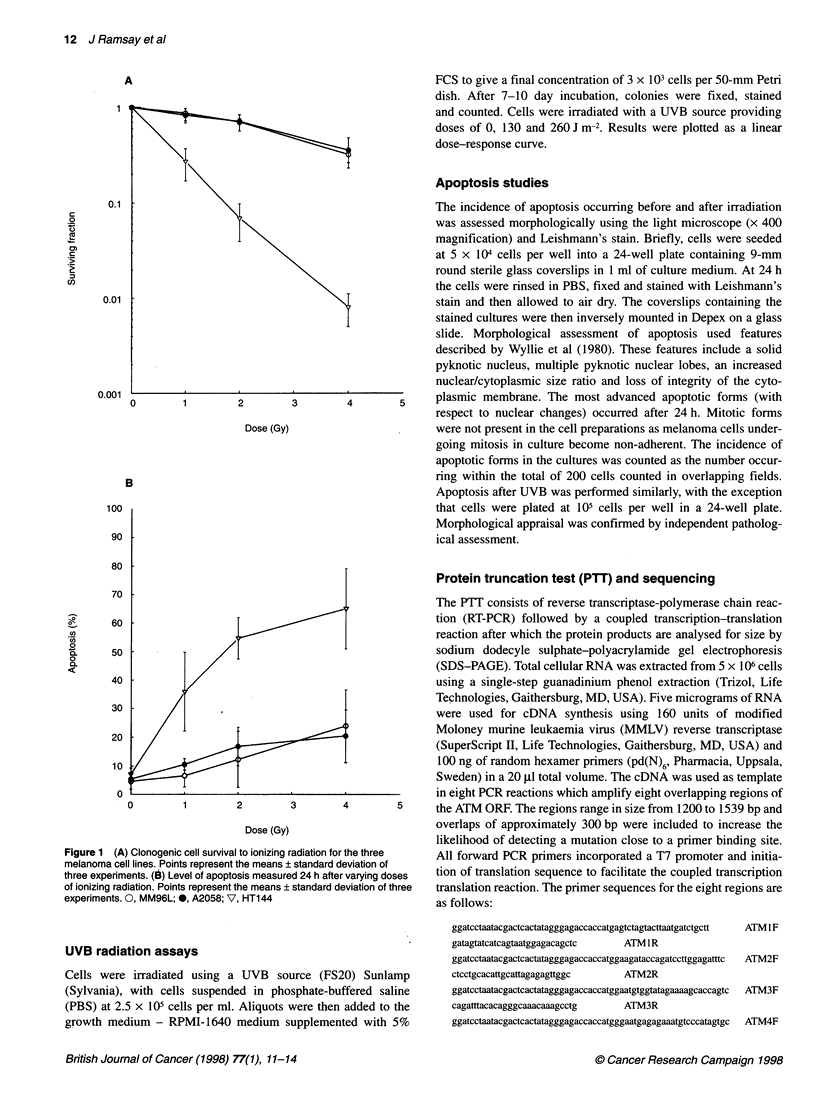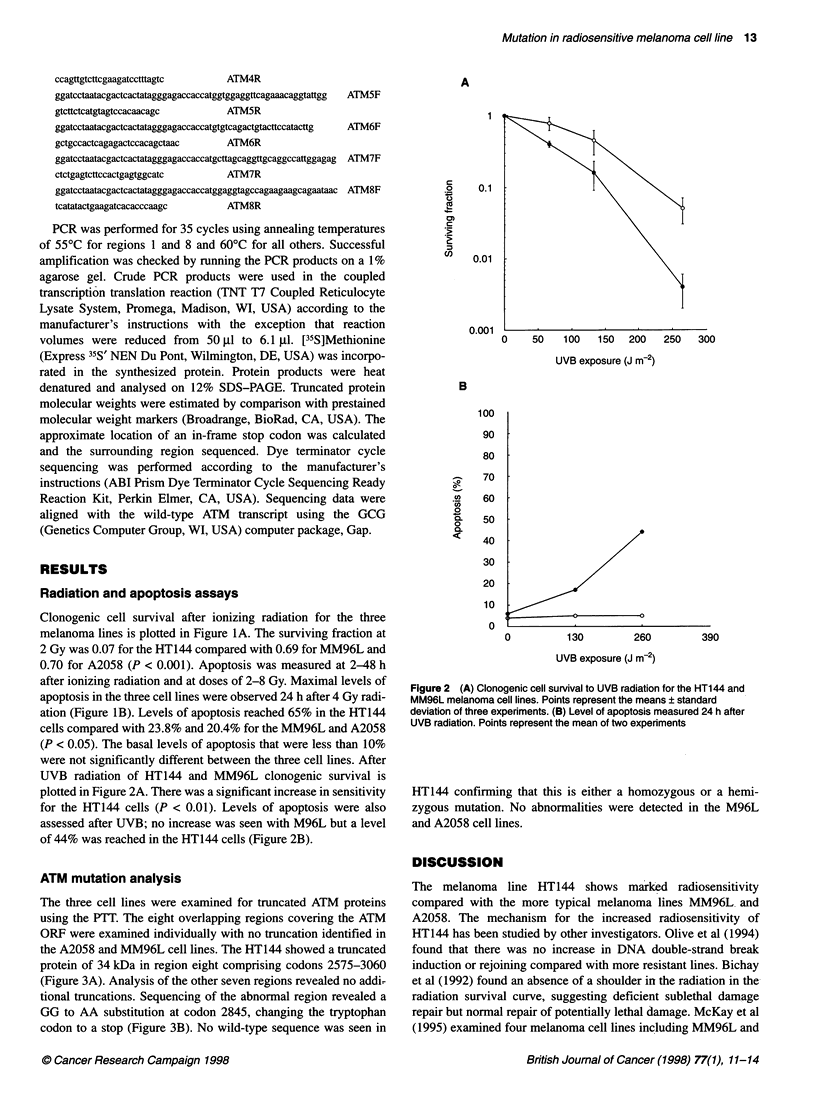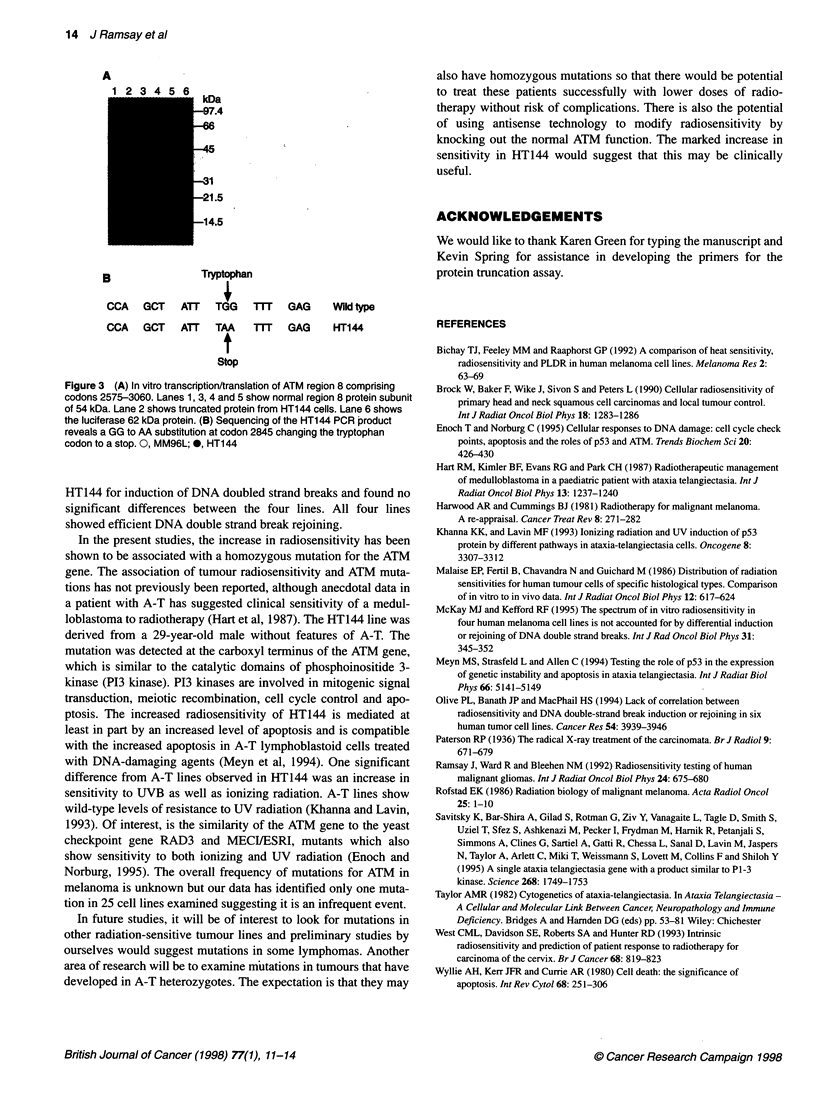# Radiosensitive melanoma cell line with mutation of the gene for ataxia telangiectasia.

**DOI:** 10.1038/bjc.1998.2

**Published:** 1998

**Authors:** J. Ramsay, G. Birrell, K. Baumann, A. Bodero, P. Parsons, M. Lavin

**Affiliations:** Queensland Radium Institute - Mater Centre, South Brisbane, Australia.

## Abstract

**Images:**


British Journal of Cancer (1998) 77(1), 11–14
© 1998 Cancer Research Campaign

### Radiosensitive melanoma cell line with mutation of the gene for ataxia telangiectasia

**J Ramsay[1,2], G Birrell[1,2], K Baumann[1], A Bodero[2], P Parsons[2] and M Lavin[2]**

[1]Queensland Radium Institute – Mater Centre, Raymond Terrace, South Brisbane, Q 4101, Australia; [2]Queensland Institute of Medical Research, Herston Rd, Herston, Q 4006, Australia

**Summary** The human melanoma cell lines MM96L, A2058 and HT144 were examined for sensitivity to ionizing radiation and UVB radiation. HT144 demonstrated a significant increase in sensitivity to ionizing and UVB radiation compared with the MM96L and A2058 cells. Sensitivity to both agents was associated with susceptibility to apoptosis. Using a protein truncation assay, a mutation for the gene for ataxia telangiectasia (ATM) was identified in HT144 cells. This was confirmed to be a homozygous mutation by subsequent sequencing of the abnormal region. Protein truncation assay of the other two cell lines showed no abnormality. The results suggest that somatic mutation of the A-T gene may be important in determining tumour radiosensitivity.

**Keywords:** melanoma; radiosensitivity; apoptosis; ataxia telangiectasia; ATM gene

From the early days of radiotherapy it has been realized that tumours vary in their sensitivity to radiation. In 1936, Paterson divided tumours into three groups: sensitive, intermediate and radioresistant. The first category included embryonic tumours and lymphomas, the second squamous cell and adenocarcinomas and the third gliomas, sarcomas and melanomas. More recently, it has been shown that this observed clinical variation in radiation response could be explained in part by differences in in vitro sensitivity of cell lines (Malaise et al, 1986). For cervical cancer (West et al, 1993), glioma (Ramsay et al, 1992) and head and neck cancers (Brock et al, 1990) there is also a wide range of in vitro radiosensitivity within each histological group and that this may correlate with clinical response (West et al, 1993). Although melanomas have been classically regarded as radioresistant, there is both clinical data (Harwood and Cummings, 1981) and in vitro data (Rofstad, 1986) to suggest that there is heterogeneity in radiation response. In this study, we have examined the mechanism in observed differences in radiosensitivity between three melanoma cell lines. An important observation has been the identification of a homozygous mutation for the gene for ataxia telangiectasia (A-T) in the radiosensitive cell line. A-T is a rare autosomal recessive condition with affected individuals showing hypersensitivity to radiation and predisposition to cancer (Taylor, 1982). Recently, the condition has been found to be caused by mutation of a single gene designated ATM (ataxia telangiectasia mutated) (Savitsky et al, 1995). Although the sensitivity of normal cells from A-T patients has been well characterized, little is known about mutations in tumours and whether this is also associated with radiosensitivity.

*Received 28 November 1996*
*Revised 2 July 1997*
*Accepted 8 July 1997*

*Correspondence to:* J Ramsay

### MATERIALS AND METHODS

### Cell lines

The established human malignant melanoma lines MM96L, A2058 and HT144 were cultured as monolayers in 5% carbon dioxide/air at 37°C in RPMI-1640 culture medium supplemented with 10% (v/v) fetal calf serum (FCS), penicillin (100 IU $ml^{-1}$), streptomycin (100 μg $ml^{-1}$) and Hepes (3 mM). The MM96L and A2058 cell lines were obtained from Dr P Parsons (Queensland Institute of Medical Research, Brisbane, Australia). The HT144 cell line was obtained from the American Type Culture Collection (Rockville, MD, USA). Cultures were routinely checked for mycoplasma contamination.

### Ionizing radiation assays

Exposure to ionizing irradiation was performed on exponentially growing cells in suspension using a 6MV linear accelerator. Cells were irradiated in standard culture medium at room temperature and were immediately returned to culture conditions. A clonogenic assay was used to assess survival and replicative potential of cells after single-fraction irradiation. Plating efficiencies were first determined to calculate the required plating densities for each cell line. Cells were plated out in 50-mm Petri dishes at densities between $10^2$ and $10^4$ cells per dish. The culture medium was 50% conditioned medium supplemented with 15% FCS. Cultures were refed at 7 days. Colonies were fixed at 10–14 days depending on growth. Colony counts were performed on cells washed in PBS then fixed and stained with crystal violet stain (5% in methanol) for 5 min. Colonies of greater than 50 cells were counted under a magnifying illuminator. The surviving fraction was calculated as the number of colonies counted divided by the number of cells plated multiplied by the inverse of the plating efficiency. Survival curves were then plotted as a common logarithmic plot.

**Figure 1** (**A**) Clonogenic cell survival to ionizing radiation for the three melanoma cell lines. Points represent the means ± standard deviation of three experiments. (**B**) Level of apoptosis measured 24 h after varying doses of ionizing radiation. Points represent the means ± standard deviation of three experiments. ○, MM96L; ●, A2058; ▽, HT144

### UVB radiation assays

Cells were irradiated using a UVB source (FS20) Sunlamp (Sylvania), with cells suspended in phosphate-buffered saline (PBS) at $2.5 \times 10^5$ cells per ml. Aliquots were then added to the growth medium – RPMI-1640 medium supplemented with 5% FCS to give a final concentration of $3 \times 10^3$ cells per 50-mm Petri dish. After 7–10 day incubation, colonies were fixed, stained and counted. Cells were irradiated with a UVB source providing doses of 0, 130 and 260 J $m^{-2}$. Results were plotted as a linear dose–response curve.

### Apoptosis studies

The incidence of apoptosis occurring before and after irradiation was assessed morphologically using the light microscope (× 400 magnification) and Leishmann's stain. Briefly, cells were seeded at $5 \times 10^4$ cells per well into a 24-well plate containing 9-mm round sterile glass coverslips in 1 ml of culture medium. At 24 h the cells were rinsed in PBS, fixed and stained with Leishmann's stain and then allowed to air dry. The coverslips containing the stained cultures were then inversely mounted in Depex on a glass slide. Morphological assessment of apoptosis used features described by Wyllie et al (1980). These features include a solid pyknotic nucleus, multiple pyknotic nuclear lobes, an increased nuclear/cytoplasmic size ratio and loss of integrity of the cytoplasmic membrane. The most advanced apoptotic forms (with respect to nuclear changes) occurred after 24 h. Mitotic forms were not present in the cell preparations as melanoma cells undergoing mitosis in culture become non-adherent. The incidence of apoptotic forms in the cultures was counted as the number occurring within the total of 200 cells counted in overlapping fields. Apoptosis after UVB was performed similarly, with the exception that cells were plated at $10^5$ cells per well in a 24-well plate. Morphological appraisal was confirmed by independent pathological assessment.

### Protein truncation test (PTT) and sequencing

The PTT consists of reverse transcriptase-polymerase chain reaction (RT-PCR) followed by a coupled transcription–translation reaction after which the protein products are analysed for size by sodium dodecyle sulphate–polyacrylamide gel electrophoresis (SDS–PAGE). Total cellular RNA was extracted from $5 \times 10^6$ cells using a single-step guanadinium phenol extraction (Trizol, Life Technologies, Gaithersburg, MD, USA). Five micrograms of RNA were used for cDNA synthesis using 160 units of modified Moloney murine leukaemia virus (MMLV) reverse transcriptase (SuperScript II, Life Technologies, Gaithersburg, MD, USA) and 100 ng of random hexamer primers (pd$(N)_6$, Pharmacia, Uppsala, Sweden) in a 20 μl total volume. The cDNA was used as template in eight PCR reactions which amplify eight overlapping regions of the ATM ORF. The regions range in size from 1200 to 1539 bp and overlaps of approximately 300 bp were included to increase the likelihood of detecting a mutation close to a primer binding site. All forward PCR primers incorporated a T7 promoter and initiation of translation sequence to facilitate the coupled transcription translation reaction. The primer sequences for the eight regions are as follows:

ggatcctaatacgactcactatagggagaccaccatgagtctagtacttaatgatctgctt ATM1F
gatagtatcatcagtaatggagacagctc ATM1R
ggatcctaatacgactcactatagggagaccaccatggaagataccagatccttggagatttc ATM2F
ctcctgcacattgcattagagagttggc ATM2R
ggatcctaatacgactcactatagggagaccaccatggaatgtggtatagaaaagcaccagtc ATM3F
cagatttacacagggcaaacaaagcctg ATM3R
ggatcctaatacgactcactatagggagaccaccatgggaatgagagaaatgtcccatagtgc ATM4F

© Cancer Research Campaign 1998

ccagttgtcttcgaagatcctttagtc ATM4R
ggatcctaatacgactcactatagggagaccaccatggtggaggttcagaaacaggtattgg ATM5F
gtcttctcatgtagtccacaacagc ATM5R
ggatcctaatacgactcactatagggagaccaccatgtgtcagactgtacttccatacttg ATM6F
gctgccactcagagactccacagctaac ATM6R
ggatcctaatacgactcactatagggagaccaccatgcttagcaggttgcaggccattggagag ATM7F
ctctgagtcttccactgagtggcatc ATM7R
ggatcctaatacgactcactatagggagaccaccatggaggtagccagaagaagcagaataac ATM8F
tcatatactgaagatcacacccaagc ATM8R

PCR was performed for 35 cycles using annealing temperatures of 55°C for regions 1 and 8 and 60°C for all others. Successful amplification was checked by running the PCR products on a 1% agarose gel. Crude PCR products were used in the coupled transcription translation reaction (TNT T7 Coupled Reticulocyte Lysate System, Promega, Madison, WI, USA) according to the manufacturer's instructions with the exception that reaction volumes were reduced from 50 µl to 6.1 µl. [$^{35}$S]Methionine (Express $^{35}$S′ NEN Du Pont, Wilmington, DE, USA) was incorporated in the synthesized protein. Protein products were heat denatured and analysed on 12% SDS–PAGE. Truncated protein molecular weights were estimated by comparison with prestained molecular weight markers (Broadrange, BioRad, CA, USA). The approximate location of an in-frame stop codon was calculated and the surrounding region sequenced. Dye terminator cycle sequencing was performed according to the manufacturer's instructions (ABI Prism Dye Terminator Cycle Sequencing Ready Reaction Kit, Perkin Elmer, CA, USA). Sequencing data were aligned with the wild-type ATM transcript using the GCG (Genetics Computer Group, WI, USA) computer package, Gap.

### RESULTS

### Radiation and apoptosis assays

Clonogenic cell survival after ionizing radiation for the three melanoma lines is plotted in Figure 1A. The surviving fraction at 2 Gy was 0.07 for the HT144 compared with 0.69 for MM96L and 0.70 for A2058 ($P < 0.001$). Apoptosis was measured at 2–48 h after ionizing radiation and at doses of 2–8 Gy. Maximal levels of apoptosis in the three cell lines were observed 24 h after 4 Gy radiation (Figure 1B). Levels of apoptosis reached 65% in the HT144 cells compared with 23.8% and 20.4% for the MM96L and A2058 ($P < 0.05$). The basal levels of apoptosis that were less than 10% were not significantly different between the three cell lines. After UVB radiation of HT144 and MM96L clonogenic survival is plotted in Figure 2A. There was a significant increase in sensitivity for the HT144 cells ($P < 0.01$). Levels of apoptosis were also assessed after UVB; no increase was seen with M96L but a level of 44% was reached in the HT144 cells (Figure 2B).

### ATM mutation analysis

The three cell lines were examined for truncated ATM proteins using the PTT. The eight overlapping regions covering the ATM ORF were examined individually with no truncation identified in the A2058 and MM96L cell lines. The HT144 showed a truncated protein of 34 kDa in region eight comprising codons 2575–3060 (Figure 3A). Analysis of the other seven regions revealed no additional truncations. Sequencing of the abnormal region revealed a GG to AA substitution at codon 2845, changing the tryptophan codon to a stop (Figure 3B). No wild-type sequence was seen in HT144 confirming that this is either a homozygous or a hemizygous mutation. No abnormalities were detected in the M96L and A2058 cell lines.

**Figure 2** (**A**) Clonogenic cell survival to UVB radiation for the HT144 and MM96L melanoma cell lines. Points represent the means ± standard deviation of three experiments. (**B**) Level of apoptosis measured 24 h after UVB radiation. Points represent the mean of two experiments

### DISCUSSION

The melanoma line HT144 shows marked radiosensitivity compared with the more typical melanoma lines MM96L and A2058. The mechanism for the increased radiosensitivity of HT144 has been studied by other investigators. Olive et al (1994) found that there was no increase in DNA double-strand break induction or rejoining compared with more resistant lines. Bichay et al (1992) found an absence of a shoulder in the radiation in the radiation survival curve, suggesting deficient sublethal damage repair but normal repair of potentially lethal damage. McKay et al (1995) examined four melanoma cell lines including MM96L and

**Figure 3** (**A**) In vitro transcription/translation of ATM region 8 comprising codons 2575–3060. Lanes 1, 3, 4 and 5 show normal region 8 protein subunit of 54 kDa. Lane 2 shows truncated protein from HT144 cells. Lane 6 shows the luciferase 62 kDa protein. (**B**) Sequencing of the HT144 PCR product reveals a GG to AA substitution at codon 2845 changing the tryptophan codon to a stop. ○, MM96L; ●, HT144

HT144 for induction of DNA doubled strand breaks and found no significant differences between the four lines. All four lines showed efficient DNA double strand break rejoining.

In the present studies, the increase in radiosensitivity has been shown to be associated with a homozygous mutation for the ATM gene. The association of tumour radiosensitivity and ATM mutations has not previously been reported, although anecdotal data in a patient with A-T has suggested clinical sensitivity of a medulloblastoma to radiotherapy (Hart et al, 1987). The HT144 line was derived from a 29-year-old male without features of A-T. The mutation was detected at the carboxyl terminus of the ATM gene, which is similar to the catalytic domains of phosphoinositide 3-kinase (PI3 kinase). PI3 kinases are involved in mitogenic signal transduction, meiotic recombination, cell cycle control and apoptosis. The increased radiosensitivity of HT144 is mediated at least in part by an increased level of apoptosis and is compatible with the increased apoptosis in A-T lymphoblastoid cells treated with DNA-damaging agents (Meyn et al, 1994). One significant difference from A-T lines observed in HT144 was an increase in sensitivity to UVB as well as ionizing radiation. A-T lines show wild-type levels of resistance to UV radiation (Khanna and Lavin, 1993). Of interest, is the similarity of the ATM gene to the yeast checkpoint gene RAD3 and MECI/ESRI, mutants which also show sensitivity to both ionizing and UV radiation (Enoch and Norburg, 1995). The overall frequency of mutations for ATM in melanoma is unknown but our data has identified only one mutation in 25 cell lines examined suggesting it is an infrequent event.

In future studies, it will be of interest to look for mutations in other radiation-sensitive tumour lines and preliminary studies by ourselves would suggest mutations in some lymphomas. Another area of research will be to examine mutations in tumours that have developed in A-T heterozygotes. The expectation is that they may also have homozygous mutations so that there would be potential to treat these patients successfully with lower doses of radiotherapy without risk of complications. There is also the potential of using antisense technology to modify radiosensitivity by knocking out the normal ATM function. The marked increase in sensitivity in HT144 would suggest that this may be clinically useful.

### ACKNOWLEDGEMENTS

We would like to thank Karen Green for typing the manuscript and Kevin Spring for assistance in developing the primers for the protein truncation assay.

### REFERENCES

Bichay TJ, Feeley MM and Raaphorst GP (1992) A comparison of heat sensitivity, radiosensitivity and PLDR in human melanoma cell lines. *Melanoma Res* **2**: 63–69

Brock W, Baker F, Wike J, Sivon S and Peters L (1990) Cellular radiosensitivity of primary head and neck squamous cell carcinomas and local tumour control. *Int J Radiat Oncol Biol Phys* **18**: 1283–1286

Enoch T and Norburg C (1995) Cellular responses to DNA damage: cell cycle check points, apoptosis and the roles of p53 and ATM. *Trends Biochem Sci* **20**: 426–430

Hart RM, Kimler BF, Evans RG and Park CH (1987) Radiotherapeutic management of medulloblastoma in a paediatric patient with ataxia telangiectasia. *Int J Radiat Oncol Biol Phys* **13**: 1237–1240

Harwood AR and Cummings BJ (1981) Radiotherapy for malignant melanoma. A re-appraisal. *Cancer Treat Rev* **8**: 271–282

Khanna KK, and Lavin MF (1993) Ionizing radiation and UV induction of p53 protein by different pathways in ataxia-telangiectasia cells. *Oncogene* **8**: 3307–3312

Malaise EP, Fertil B, Chavandra N and Guichard M (1986) Distribution of radiation sensitivities for human tumour cells of specific histological types. Comparison of in vitro to in vivo data. *Int J Radiat Oncol Biol Phys* **12**: 617–624

McKay MJ and Kefford RF (1995) The spectrum of in vitro radiosensitivity in four human melanoma cell lines is not accounted for by differential induction or rejoining of DNA double strand breaks. *Int J Rad Oncol Biol Phys* **31**: 345–352

Meyn MS, Strasfeld L and Allen C (1994) Testing the role of p53 in the expression of genetic instability and apoptosis in ataxia telangiectasia. *Int J Radiat Biol Phys* **66**: 5141–5149

Olive PL, Banath JP and MacPhail HS (1994) Lack of correlation between radiosensitivity and DNA double-strand break induction or rejoining in six human tumor cell lines. *Cancer Res* **54**: 3939–3946

Paterson RP (1936) The radical X-ray treatment of the carcinomata. *Br J Radiol* **9**: 671–679

Ramsay J, Ward R and Bleehen NM (1992) Radiosensitivity testing of human malignant gliomas. *Int J Radiat Oncol Biol Phys* **24**: 675–680

Rofstad EK (1986) Radiation biology of malignant melanoma. *Acta Radiol Oncol* **25**: 1–10

Savitsky K, Bar-Shira A, Gilad S, Rotman G, Ziv Y, Vanagaite L, Tagle D, Smith S, Uziel T, Sfez S, Ashkenazi M, Pecker I, Frydman M, Harnik R, Petanjali S, Simmons A, Clines G, Sartiel A, Gatti R, Chessa L, Sanal D, Lavin M, Jaspers N, Taylor A, Arlett C, Miki T, Weissmann S, Lovett M, Collins F and Shiloh Y (1995) A single ataxia telangiectasia gene with a product similar to P1-3 kinase. *Science* **268**: 1749–1753

Taylor AMR (1982) Cytogenetics of ataxia-telangiectasia. In *Ataxia Telangiectasia – A Cellular and Molecular Link Between Cancer, Neuropathology and Immune Deficiency*. Bridges A and Harnden DG (eds) pp. 53–81 Wiley: Chichester

West CML, Davidson SE, Roberts SA and Hunter RD (1993) Intrinsic radiosensitivity and prediction of patient response to radiotherapy for carcinoma of the cervix. *Br J Cancer* **68**: 819–823

Wyllie AH, Kerr JFR and Currie AR (1980) Cell death: the significance of apoptosis. *Int Rev Cytol* **68**: 251–306

© Cancer Research Campaign 1998